# Therapeutic Potential of Propolis and Royal Jelly in *Encephalitozoon Intestinalis* Infection: An in Vitro Study

**DOI:** 10.1007/s11686-024-00956-2

**Published:** 2025-01-24

**Authors:** Derya Gül Gülpinar, Zübeyda Akın Polat, Ülfet Çetinkaya

**Affiliations:** 1 Departments of Medical Parasitology, Cumhuriyet University School of Medicine, Sivas, Türkiye; 2 Genkök Genome and Stem Cell Center, Erciyes University, Talas, Kayseri, 38039 Türkiye; 3 Halil Bayraktar Health Vocational High School, Erciyes University, Talas, Kayseri, 38039 Türkiye

**Keywords:** *Encephalitozoon intestinalis*, Propolis, Royal jelly, In vitro

## Abstract

**Purpose:**

Encephalitozoon intestinalis is an obligate intracellular microsporidian fungus that causes severe gastrointestinal infections, particularly in immunocompromised individuals. Propolis (PROP), a resinous substance derived from bees, has antimicrobial, anti-inflammatory and antioxidant properties, while royal jelly (RJ) has immunomodulatory, antioxidant and antimicrobial activities. The aim of this study was to investigate the therapeutic potential of PROP and RJ against E. intestinalis.

**Methods:**

The phenolic composition of PROP was analysed by high-performance liquid chromatography with diode array detection, and the chemical components of RJ were evaluated according to ISO12824 standards. The cytotoxicity of PROP and RJ on HEK-293 cells was evaluated using the XTT assay. The three highest non-cytotoxic concentrations of each sample were tested for their effects on E. intestinalis spores by qRT-PCR. Trichrome-stained photomicrographs were used to assess spore density in HEK-293 cells treated with PROP and RJ.

**Results:**

PROP analysis revealed flavonoids such as quercetin, kaempferol, pinocembrin and galangin, as well as phenolic acids such as caffeic and cinnamic acids, known for their bioactive properties. RJ contained mainly proteins, lipids, carbohydrates and sugars, reflecting its role as a nutritionally and biologically active substance. According to the results of this first study evaluating the effect of PROP and RJ on E. intestinalis, all concentrations evaluated in the study showed a significant inhibitory effect on the growth of E. intestinalis spores compared to the control group.

**Conclusion:**

In conclusion, we believe that PROP and RJ should be considered as an alternative option in the development of antimicrosporidial drugs due to their potential medicinal and pharmaceutical properties.

## Introduction

Microsporidia are obligate unicellular, intracellular spore-forming parasitic fungi grouped with Cryptomycota [1]. Microsporidia have a wide host range from protists to vertebrates [2]. Of the approximately 1500 species identified, 17 are pathogenic to humans. *Enterocytozoon bieneusi* and *Encephalitozoon intestinalis* are the 2 most commonly detected microsporidian [3]. *Encephalitozoon* species have the ability to disseminate widely within their hosts, potentially affecting almost all organ systems during infection. *E. intestinalis* infections primarily manifest as gastrointestinal disease, particularly chronic diarrhoea and malabsorption syndrome, but in immunocompromised individuals can progress to disseminated infections involving organs such as the liver, kidneys, lungs and eyes [4, 5]. These infections are predominantly observed in immunocompromised patients, including those with HIV and CD4 counts < 50/mm³, transplant recipients and those undergoing immunomodulatory therapy. However, *Encephalitozoon* infections have also been documented in immunocompetent individuals [6, 7]. Treatment of infections caused by microsporidian species is not easy because they are intracellular pathogens and show natural resistance due to their spore wall structure [8, 9]. Albendazole, which inhibits tubulin, and fumagillin, which inhibits methionine aminopeptidase type 2 (MetAP2), are the two main therapeutic agents used to treat microsporidiosis [10]. Due to the failure of existing drugs to treat microsporidiosis infections, new therapeutic agents have been developed.

Propolis (PROP) is a resinous substance produced by honeybees by mixing their salivary gland secretions with exudate accumulated from various parts of plants, especially branches, bark, flower buds, leaves and stems. It is a bee product that has been used in medicine since ancient times [11, 12]. Although its anti-inflammatory, antioxidant, antimicrobial, antitumour and immunomodulatory activities have been studied, research on its antiparasitic properties is limited [13, 14]. Royal jelly (RJ) is produced by the hypopharyngeal and mandibular glands of worker bees and plays a critical role in the development of queen bees and larvae [15]. As a natural and highly nutritious product, RJ has great potential for use in medicine, cosmetics and as a health-promoting food. RJ exhibits numerous biological activities, including vasodilatory, hypotensive, antihypercholesterolemic, anti-diabetic, immunomodulatory, anti-inflammatory, antioxidant, anti-aging, neuroprotective, antimicrobial, estrogenic, anti-allergic, anti-osteoporotic and anti-tumour effects [16–18].

The aim of this study was to evaluate the in vitro effects of PROP and RJ on *Encephalitozoon intestinalis* the most common fungus in humans, due to the lack of similar studies in the literature. These natural products are expected to contribute to the development of promising new therapeutic agents for the treatment of microsporidiosis.

## Materials and methods

### PROP and RJ Samples and Analysis

The PROP and RJ samples used in this study were obtained from Eğriçayır Organic Bee Products Limited Company. The phenolic content of PROP was analysed by high performance liquid chromatography with diode array detection (HPLC-DAD). The chemical composition of RJ was evaluated according to the standards of the International Organisation for Standardisation (ISO) 12,824.

For propolis extraction, raw propolis samples were frozen at -20 °C for 24 h, then crushed into small pieces and ground to a fine powder using a mill. The powdered propolis was mixed with 70% ethanol for extraction. The mixture was stirred at room temperature for 48 h using a magnetic stirrer and then filtered through Whatman No. 1 filter paper (Whatman^®^ No. 1, Buckinghamshire, UK). The filtrate was further filtered through a polyvinylidene difluoride syringe filter (Millipore Millex-HV, 0.45 μm) and transferred to 1.5 mL vials for injection into the HPLC-DAD system. Chromatographic separation was performed on an Inertsil^®^ ODS-3 column (5 μm, 4.6 × 150 mm, Tokyo, Japan) at a flow rate of 1 mL/min. The mobile phases consisted of mobile phase A (0.1% formic acid in deionised water, v/v) and mobile phase B (acetonitrile). The analysis was performed at a column temperature of 30 °C, with an injection volume of 5 µL, a detection wavelength of 270 nm and a gradient system. Stock solutions of phenolic compounds were prepared at a concentration of 1 mg/mL in methanol. Standards of phenolic compounds were injected into the HPLC system to identify the main peaks, retention times and spectra.

For the preparation of RJ concentrations, 1000 mg of RJ was homogenised with 10 mL of colourless DMEM medium. The mixture was centrifuged at 6000 rpm for 40 min. The clear supernatant was collected and transferred to a new centrifuge tube. To the pellet, 10 mL of DMEM medium containing 1% dimethylsulfoxide (DMSO) was added and the suspension was centrifuged again at 6000 rpm for 40 min. The second supernatant was collected, combined with the first supernatant and filtered through a 0.22 μm pore filter to give a sterile stock solution with a final concentration of 50 mg/mL. Serial dilutions were then prepared from this stock solution, ranging from 50 mg/mL to 0.09 mg/mL. The chemical composition of RJ was analysed by Intertek Testing Services in accordance with the chemical requirements of ISO 12,824 (Reference number ISO 12824:2016).

### Cytotoxic Potential of PROP and RJ

Cytotoxicity tests were performed using HEK-293 cells (human renal epithelial cells, ATCC) [19]. Cells were cultured in Dulbecco’s minimum Eagle’s medium (DMEM) containing 10% fetal bovine serum (FBS) and 1% penicillin-streptomycin solution. Cultures were maintained in a humidified incubator at 37 °C with 5% CO_2_. The cytotoxicity of PROP and RJ was evaluated in HEK-293 cells using the XTT assay. Cells were seeded in 96-well plates at a density of 1 × 10⁴ cells/mL (100 µL per well) and incubated for 24 h to allow cell attachment. After attachment, 100 µL of final solutions prepared in serial dilutions at concentrations of 1000–1.9 µg/mL for PROP and 50-0.009 mg/mL for RJ were added to the cultures and incubated again for 24 h. Then 50 µL of XTT reagent was added to each well and after two hours the absorbance was measured at 450 nm using a microplate reader. Control wells contained cells without samples and the basal control contained only culture medium and XTT reagent. Cytotoxicity was assessed in quadruplicate and cell viability (%) was calculated as [(OD450(sample)/OD450(negative control)) × 100].

### Assessment of the Effect of PROP and RJ on E. Intestinalis

#### E. Intestinalis Strain and Preparation of Spores

**Fig. 1:**
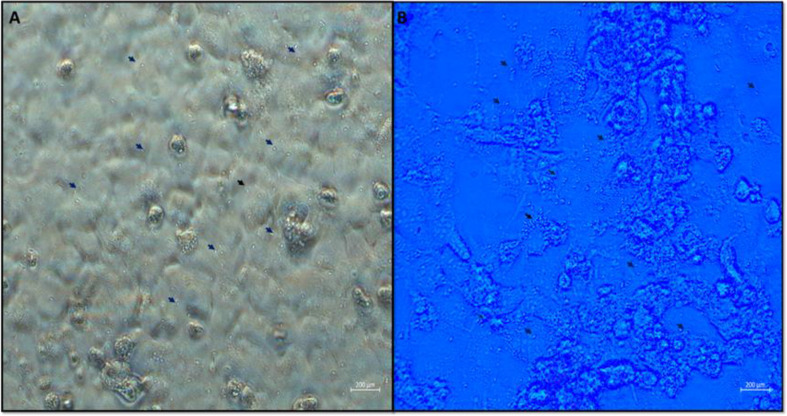
Unfiltered (**A**) and blue-filtered (**B**) views of free *E. intestinalis* spores (arrows) produced in the HEK-293 cell line (×40)

The HEK-293 cell line was used to obtain *E. intestinalis* spores (ATCC 50506). Cells were seeded in 25 cm^3^ tissue culture plates and incubated at 37 °C in 5% CO_2_ medium. Washings with PBS were performed to remove non-attached cells. *E. intestinalis* spores were added, at a ratio of 5 parasites/1 cell. and incubated at 37 °C in 5% CO_2_ overnight for infection. The next day, the plates were washed with PBS to remove spores that had not entered the cells. Ten days after spore inoculation, free spores were observed in the medium (Fig. 1). Twice a week, free spores in the medium were collected, passed through a 5 μm porous filter to remove dead cells and centrifuged at 4000 rpm for 15 min to separate the spores and stored at + 4◦C.

#### *Evaluation of E. Intestinalis Load by Quantitative Real Time Polymerase Chain Reaction* (*qRT-PCR Method)*

##### Infection of Host Cells and Treatment with PROP and RJ

HEK-293 cells were cultured in 6-well plates at a density of 3 mL per well, with 1 × 10^5^ cells per well. Medium containing 5 × 10^5^ spores was added and incubated to initiate infection. After infection, DMEM medium supplemented with 2% FBS and the three highest non-cytotoxic concentrations of PROP (250, 125, 62.5 µg/mL) and RJ (25, 12.5, 6.25 mg/mL) was added. At this stage, in contrast to the medium used in the cytotoxic potential of PROP and RJ, 2% FBS was used to stress the cells, slow down their proliferation and infect them with spores. Cultures were maintained in an incubator at 37 °C with 5% CO_2_. Proliferation of HEK cells, the host cell of *E. intestinalis* spores, leads to depletion of essential nutrients in the medium and pH changes as a result of cellular metabolism. For these reasons, the medium was renewed on days 3 and 7 of the 10-day incubation period using the same concentrations of PROP and RJ. The experiment was terminated 10 days after infection. To assess *E. intestinalis* density, all media and trypsinised host cells were collected separately. These were then centrifuged at 4000 rpm for 15 min. The supernatant was removed, and the resulting pellet was resuspended in medium and stored at -20 °C for subsequent DNA extraction.

##### DNA Extraction

Samples were stored at -20 °C and thawed at room temperature prior to DNA isolation. DNA isolation was performed using the PureLink Genomic DNA Mini Kit (Invitrogen, USA) according to the recommended protocol of the kit. Briefly, digestion buffer and 20 µL Proteinase K were added to each sample, followed by vortexing. Samples were incubated overnight at 55 °C. After incubation, the lysate was centrifuged at room temperature to remove particles and 20 µL of RNase A was added to the supernatant. After this treatment, the mixture was incubated again. After incubation, 200 µL of Lysis/Binding Buffer was added to the sample and vortexed. Then 200 µL of 96–100% ethanol was added and mixed. The resulting 640 µL lysate was transferred to a spin column and centrifuged. The collection tube was removed, the spin column was placed in a new collection tube and washed according to the kit protocol. The isolated DNA was stored at -20 °C until use.

##### qRt-PCR Method

Specific primers targeting the 16S small subunit rRNA sequence were used: F (5’ - CCTGACTGGACGGACAGAAG − 3’) and R (5’ - TTCGTCCTTCATCGTCACAT − 3’) [20]. Amplification was performed in a total reaction volume of 20 µl, containing 3 µl distilled water, 10 µl 2x SYBR-Green Master Mix (Roche Diagnostic, Germany), 1 µl of each primer (10 pmol) and 5 µl extracted DNA. The thermal cycling conditions were as follows: initial denaturation at 95 °C for 5 min, followed by 45 cycles of 95 °C for 10 s, 60 °C for 10 s and 72 °C for 10 s. This was followed by a single cycle of 95 °C for 5 s, 65 °C for 1 min and a final step at 40 °C for 30 s. A dilution series of plasmids encoding the *E. intestinalis* 16 S SSU rRNA region served as a positive control, with a calculated copy number of 2.8 × 10^8^ in the main stock. This stock was diluted tenfold to produce nine sequential dilutions. Sterile distilled water was used as a negative control. All experiments were performed in four replicates.

### Visualisation of the Effects of PROP and RJ on E. Intestinalis Spores in HEK-293 Cells

To assess the effect of PROP and RJ on *E. intestinalis* spores, sterile coverslips were placed at the bottom of 6-well tissue culture plates, with three replicates per sample. The procedures described in the section “Assessment of the effect of PROP and RJ on *E. intestinalis*” were followed. After 10 days of incubation, the coverslips were gently removed, stained with trichrome and examined at ×100 magnification, with detailed photographic documentation.

### Statistical Analysis

The data collected, revised and validated to assess the in vitro effects of PROP and RJ were analysed using the Statistical Package for Social Sciences (SPSS) version 23. Statistical analysis was based on four replicates, with results expressed as mean ± standard deviation (SD). Spore counts for PROP and RJ concentration were compared with the control groups using an independent two-sample t-test. One-way ANOVA was used to compare mean *E. intestisnalis* spore counts across PROP and RJ concentrations. Pairwise comparisons of PROP and RJ concentrations were made using Tukey’s post-hoc test.

## Results

### Analysis of PROP and RJ Samples

As a result of the analysis, flavonoids such as galangin, quercetin, kaempferol, epigallocatechin gallate from the flavonol group; pinocembrin and naringenin from the flavanone group; apigenin and chyricin from the flavone group; trans-chalcone from the chalcone group were detected in the PROP sample. Galangin was the flavonoid detected in the highest concentration (3843.63 µg/g). In addition, non-flavonoid compounds such as caffeic acid, p-coumaric acid, trans-ferulic acid, trans-isoferulic acid, dimethoxycinnamic acid, trans-cinnamic acid, caffeic acid phenethyl ester (CAPE) were detected. CAPE was the non-flavonoid detected at the highest concentration (5157.48 µg/g). In the analysis of PROP sample, 17 phenolic compounds were determined. The amounts, classification, biological and pharmacological activity of these compounds are listed in Table 1.

**Table 1:** Classification, amount, Biological and pharmacological activity of phenolic component content of PROP

Phenolic Compounds	Classification	Biological and Pharmacological Activity	Amount (µg/g)
Gallic acid	Non-flavonoid Hydroxybenzoic acids	Anti-inflammatory, Cardioprotective effect, Antineoplastic activity, Metabolic disease, urogenital disease, respiratory disease [21–26]	189.17
Epigallocatechin gallate	Flavonoid Flavanol	antioxidant, antibacterial, antifungal, anti-cardiovascular, and antiviral [27–29]	11.04
Caffeic acid	Non-flavonoid Hydroxycinnamic acid	anticarcinogenic activity, antiinflammatory, anti-oxidant activity, medications and cosmetic use [30–33]	461.85
P-Coumaric acid	Non-flavonoid Hydroxycinnamic acid	UV protective, hypopigmentation and antimelanogenic effect, immunomodulatory and antiinflammatory activity, antidiabetic and antihyperlipidemic activity [34–36]	12.96
Trans-Ferulic acid	Non-flavonoid Hydroxycinnamic acid	anti-inflammatory, anti-aging, antiangiogenic, anticancer, antimicrobial, and antioxidant [37]	185.19
Trans-Isoferulic acid	Non-flavonoid Hydroxycinnamic acid		26.43
Dimethoxycinnamic acid	Pheonolic component	antioksidan, anti-inflamatuar [38]	149.73
Quercetin	Flavonoid Flavonol	antioxidant, anti-inflammatory, anti-cardiovascular, antibacterial [39–41]	280.35
Trans-Cinnamic acid	Pheonolic component	Antioxidant, antitumor, antimicrobial [42–44]	84.84
Naringenin	Flavonoid Flavanones	antioxidant, anti-inflammatory, anti-cardiovascular, antiviral [45–47]	752.01
Apigenin	Flavonoid Flavone	antiviral, anti-hyperglycemic, antioxidant, anti-inflammatory [48–51]	270.51
Kaempferol	Flavonoid Flavonol	antioxidant, antimicrobial, anticancer, neuroprotective, hepatoprotective activity [52]	177.90
Chrysin	Flavonoid Flavone	antitumor, anti-inflammatory, antiviral and antioxidant [53]	1203.75
Pinocembrin	Flavonoid Flavanone	antimicrobial, anti-inflammatory, antioxidant, and anticancer [54]	2811.00
Galangin	Flavonoid Flavonol	antifungal, anti-inflammatory, antiviral, antibacterial, and antidiabetic [55]	3843.63
Caffeic acid phenethyl ester (CAPE)	Pheonolic component	anti-tumor, anti-oxidation, anti-inflammatory, immune regulation [56]	5157.48
Trans-Chalcon	Flavonoid chalcones	anti-inflammatory and anticancer	2640.54

Proteins, lipids, carbohydrates, lipid content and sugars were generally found in the structure of the analysed RJ sample (Table 2). The analyses revealed the presence of a substance called 10-hydroxy-α-2-deconoic acid (10-HAD), which has antibiotic activity against many bacteria and fungi [57].The amount of 10-HAD, which is one of the most important quality factors for RJ, was determined to be 2.39%.

**Table 2:** Chemical composition of RJ

Content	Amount-Unit
Moist content	63.3%
10-Hydroxy-2-Decanoic acid (10-HDA)	2.39%
Protein	14.6%
Total sugar	11.7%
Fructose	5.2%
Glucose	4.8%
Sucrose	1.7%
Total acidity (mL 1 N NaOH/100 g)	36.5%
Total lipids	3.3%

### Cytotoxic Potential of PROP and RJ

**Fig. 2:**
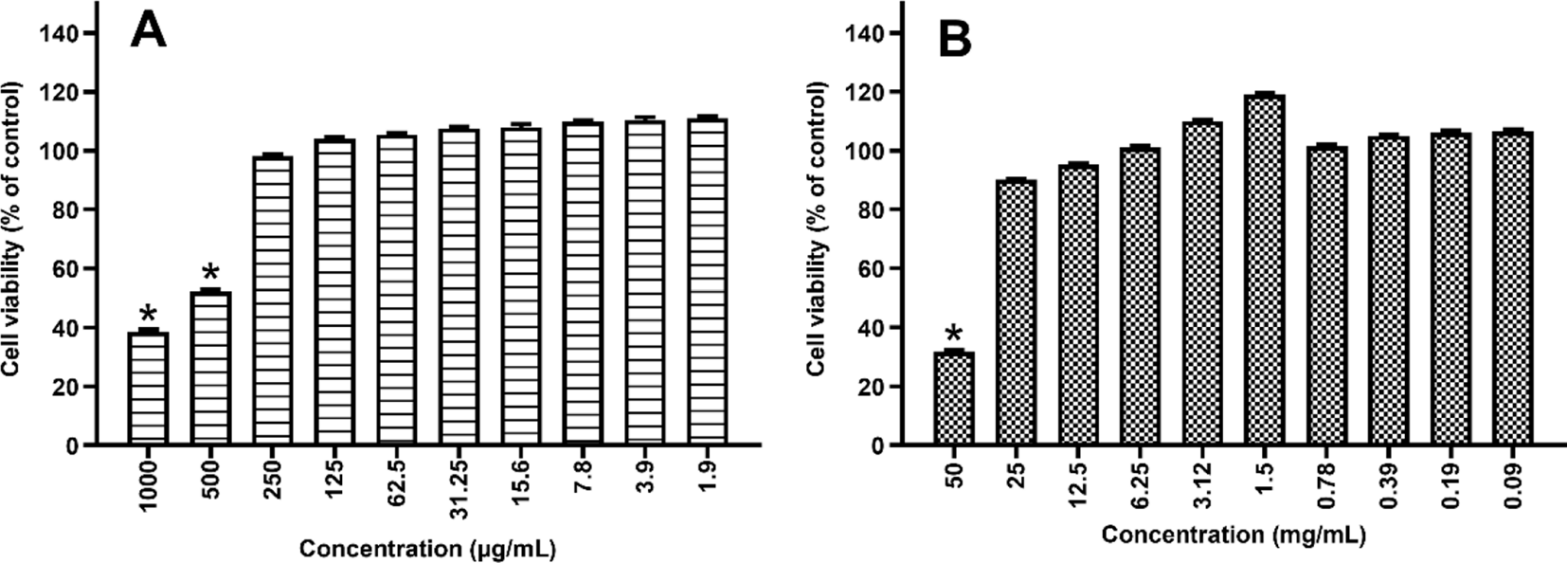
PROP (1000, 500, 250, 125, 62.5, 31.25, 15.6, 7.8, 3.9, 1.9 µg/mL) (**A**) and RJ (50, 25, 12.5, 6.25, 3.12, 1.5, 0.78, 0.39, 0.19, 0.09 mg/mL) (**B**) in vitro cytotoxicity. ^*^p < 0.05 vs. control

According to the cytotoxicity results, 250, 125, 62.5 µg/mL concentrations of PROP did not show toxic effect on HEK-293 cells (*p* > 0.05); however, the difference in viability rate of 1000, 500 µg/mL concentrations was statistically significant (*p* < 0.05) compared to the control, these concentrations were considered as cytotoxic (Fig. 2.A.). RJ concentrations of 25, 12.5, 6.25 mg/mL showed no toxic effect on HEK-293 cells (*p* > 0.05) (Fig. 2.B).

### Evaluation of the E. Intestinalis Spore Load of PROP and RJ

**Fig. 3:**
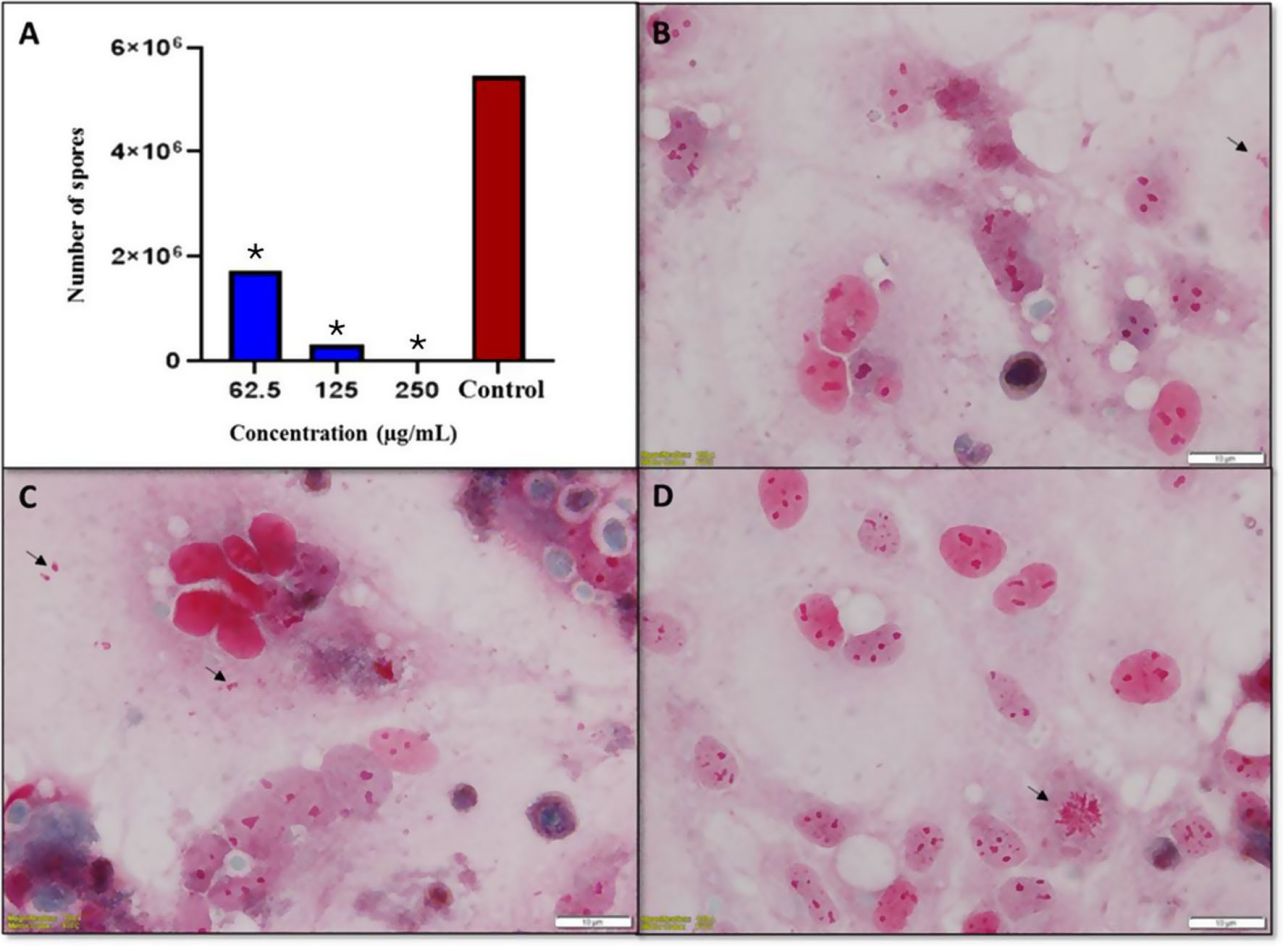
(**A**) qRT-PCR results of the in vitro effect of PROP concentrations (250 µg/mL, 125 µg/mL and 62.5 µg/mL) on *E. intestinalis* spores after 10 days of incubation.*p < 0.05 vs. control. (**B**-**D**) Trichrome staining images showing *E. intestinalis* spore density at PROP concentrations of 250 µg/mL (**B**), 125 µg/mL (**C**) and 62.5 µg/mL (**D**) after 10 days of incubation. Arrows indicate free and intracellular spores and highlight differences in spore density between the concentrations applied

All concentrations of PROP evaluated showed a significant inhibitory effect on the growth of *E. intestinalis* spores compared to the control group (*p* < 0.05). The three highest non-cytotoxic concentrations of PROP evaluated in vitro, 250, 125 and 62.5 µg/mL, showed 99.62%, 94.27% and 68.4% inhibition, respectively (Fig. 3).

**Fig. 4:**
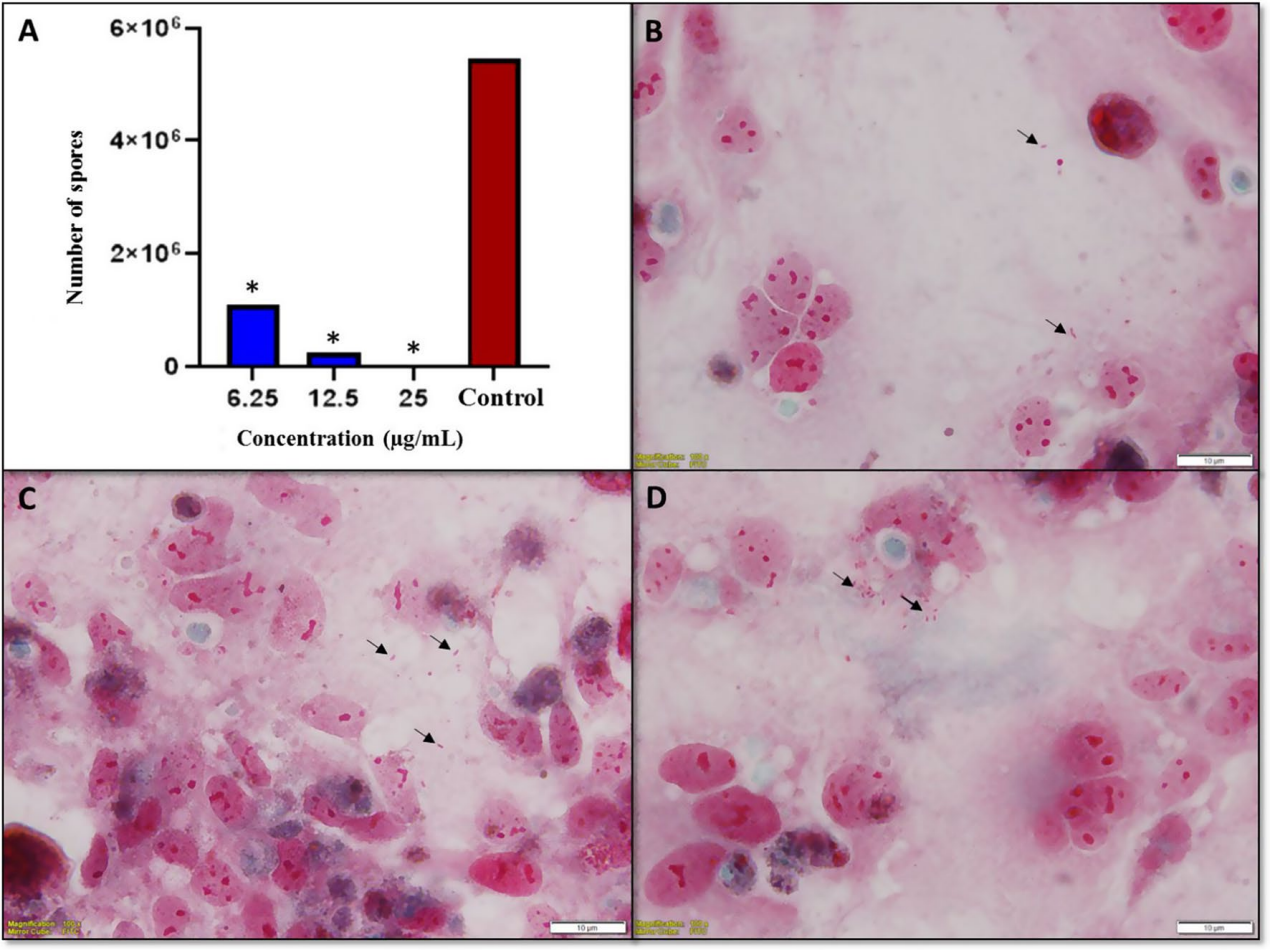
(**A**) qRT-PCR analysis demonstrating the in vitro effects of RJ at concentrations of 25 mg/mL, 12.5 mg/mL and 6.25 mg/mL on *E. intestinalis* spores after 10 days of incubation (*p < 0.05 vs. control). (**B**-**D**) Trichrome-stained images showing the spore density of *E. intestinalis* at PROP concentrations of 25 mg/mL (**B**), 12.5 mg/mL (**C**) and 6.25 mg/mL (**D**) after 10 days of incubation. Arrows indicate free and intracellular spores, highlighting the variation in spore density at different concentrations

Similar to the PROP results, all concentrations of RJ had an inhibitory effect on the growth of *E. intestinalis* spores in comparison to the control group (*p* < 0.05). The three highest non-cytotoxic concentrations of RJ, 25, 12.5, 6.25 mg/mL, showed 99.35%, 95.5%, 80.16% antimicrosporidial effect, respectively (Fig. 4).

**Fig. 5:**
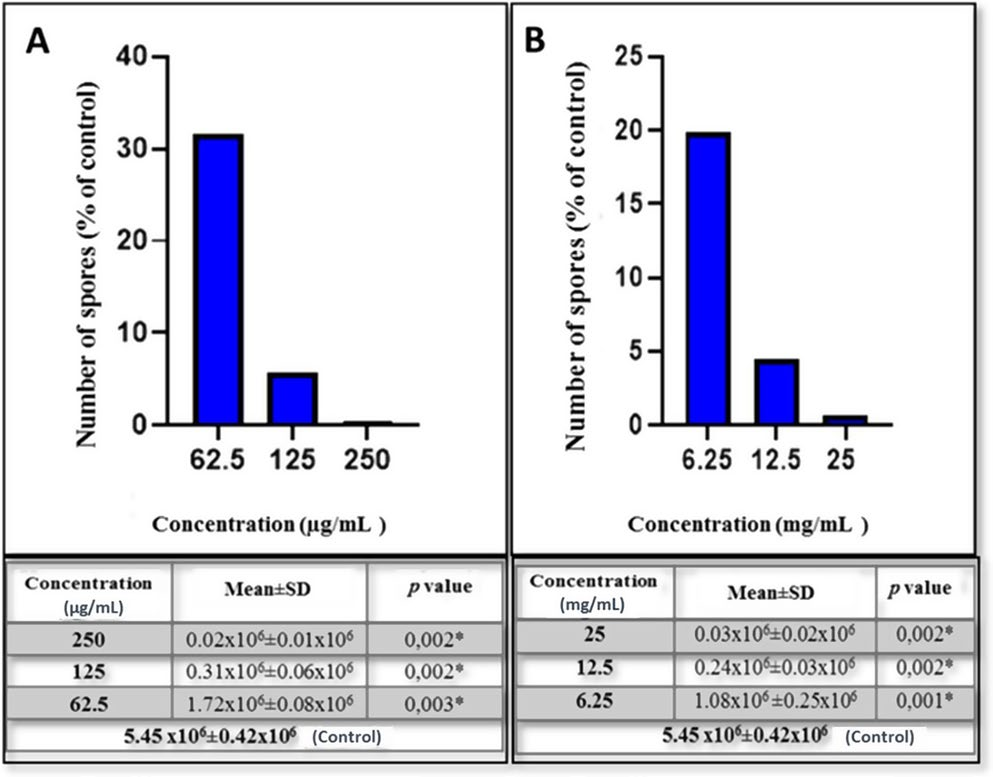
Percentage viability rates, means and p-values of qRt-PCR data on the effects of PROP (**A**) and RJ (**B**) concentrations used in the study on *E. intestinalis* spores versus control

The results indicate that both PROP and RJ have a dose-dependent inhibitory effect on the growth of *E. intestinalis* spores, as confirmed by independent two-sample t-tests (*p* < 0.05). The detailed percentage viability rates, mean ± SD values and p values for the qRt-PCR data are shown in Fig. 5, highlighting the comparative effects of PROP and RJ concentrations on *E. intestinalis* spore viability versus control.

## Discussion

Microsporidiosis is a common infection in AIDS and immunocompromised individuals worldwide, and its prevalence and severity have increased significantly in recent years [58]. The most common manifestation is gastrointestinal infection, but it can infect almost any organ system [59]. Various medical regimens have been used to treat microsporidiosis, but treatment results range from disappointing to promising [10]. Our study provides the first evaluation of PROP and RJ on *E. intestinalis* spores in the literature.

PROP is a bee product that has been used in medicine since ancient times. Studies have shown that PROP has two distinct effects in terms of its antimicrobial and antiparasitic properties, namely its effect on the pathogen and its effect on the host [60]. With regard to pathogens, PROP acts by inhibiting the ability of pathogens to invade host cells (by forming a physical barrier and inhibiting enzymes and proteins required for invasion of host cells) [60, 61]. PROP also inhibits the replication process of pathogens by inhibiting the enzymes required to replicate the genetic material of pathogens [62, 63]. For the host, PROP acts as an immunomodulator [64–66]. It regulates innate immunity and modulates inflammatory pathways [60, 67]. PROP also helps to maintain the host’s cellular antioxidant status throughout infection [68].

The curative properties of PROP and RJ are directly related to their chemical constituents. However, the chemical constituents of bee products are complex and regional variations in their antiparasitic activity may differ according to their botanical source and geographical origin [69–71]. Therefore, the chemical constituents of PROP and RJ used in this study were characterised and evaluated.

Although HEK-293 cells are widely used in biotechnology and cell biology [72]; the selection of this cell line was affected by several factors. First of all, HEK293 cells are a well-characterized, easily cultured cell line. In addition, *E. intestinalis* causes widespread infection in immunocompromised patients and can cause renal involvement in these patients [73]. In the study conducted by Çetinkaya and colleagues on cells that can be host cells for *E. intestinalis* species under in vitro conditions, they found that the reproductive potential of *E. intestinalis* in HEK-293 cells was quite high and the number of spores increased significantly [74]. Therefore, in the study, HEK-293 cell line was used as a host cell to evaluate the therapeutic potential of PROP and RJ on *E. intestinalis* and cytotoxicity tests were performed on these cells.

According to the results of HPLC-DAD analysis of PROP, the most dominant phenolic compounds were found to be caffeic acid, phenethyl ester, galangin, pinocembrin, trans-chalcone, chrysin, naringenin. Smaller amounts of gallic acid, epigallocatechin gallate, p-coumaric acid, trans-ferulic acid, trans-isoferulic acid, dimethoxycinnamic acid, quercetin, transcinnamic acid, apigenin, kaempferol. The constituents found are similar to those reported in the literature [75–77].

Apigenin, quercetin and caffeic acid exhibit antiparasitic effects through different mechanisms of action. Studies found that apigenin induced inhibition of cell proliferation and upregulation of reactive oxygen species (ROS) expression in *L. amazonensis* and altered the mitochondrial membrane potential of the parasite by causing swelling in parasite mitochondria [78]. In the *L. amazonensis* study, quercetin treatment significantly increased ROS production and induced mitochondrial function and membrane potential disturbances [79]. Caffeic acid caused morphological changes in parasitic cells, integrity of the cellular plasma membrane and mitochondria, and promoted apoptosis. Microsporidia are obligate intracellular pathogens. It takes about 1 week to multiply in the infected cell. During this period, the infected cell swells and reaches about twice its size. Under normal conditions, microsporidia species complete the life cycle by preventing the cell from entering apoptosis while growing cells go to apoptosis [80]. We think that the caffeic acid in PROP encourages the cells to apoptosis and the spore density decreases because *E. intestinalis* cannot complete its life cycle. However, studies have shown that caffeic acid enhanced the inflammatory response of infected macrophages by promoting the expression of ROS and TNF-α, as well as decreasing the expression of IL-10 and the presence of iron, which significantly increased the antiparasitic activity of macrophages [81]. Özdal et al. [82] reported that the caffeic acid content in PROP samples collected from different parts of Türkiye ranged from 0.04 to 0.61 mg/g. In our study, caffeic acid was found between these values in the analytical results of PROP. According to our HPLC-DAD analysis results, CAPE (5157.48 µg/g) was the phenolic component detected in the highest amount in the ethanolic extract of propolis and it was reported that this component exhibited potential antifungal, antibiofilm, anti-tumor, anti-oxidation, anti-inflammatory, immune regulation properties [56, 83]. According to the results of the analysis, galangin (3843.63 µg/g), which was detected in the highest amount among the flavonols, shows many pharmacological properties including antifungal anti-inflammatory, antiviral, antibacterial, and antidiabetic activities [55]. The biological and pharmacological activities of phenolic compounds may be attributed to the antimicrosporidial activity of PROP. In the literature, some studies evaluating the effect of PROP on microsporidiosis in bees infected with Microsporidia have been reported. Ethanol extracts of PROP significantly reduced *Nosema ceranae* infection rate and bee mortality [84].A dichloromethane extract of PROP from northern New York was reported to greatly reduce *N. ceranae* spore loads in a dose-dependent manner [85]. In a similar study, ethanol extract of PROP from honey bee *Apis mellifera* in Italy was reported to reduce spore load, increase food consumption and survival of worker bees infected with *N. ceranae* [86].

There is a wide range of extraction methods used to obtain PROP extracts. They range from the traditional separation technique using an organic solvent such as ethanol to more complex methods such as supercritical fluid extraction. Extraction methods can affect the amount of active ingredient in the extract. Therefore, it can alter the biological activity of extracts [87, 88]. The reason why ethanol is mostly preferred in PROP extraction in industry is that it dissolves more bioactives [89]. For this reason, ethanol was used as a solvent in the preparation of PROP concentrations in our study due to the high biological activity of ethanolic PROP extracts.

In our study, a significant inhibitory effect of PROP was observed on *E. intestinalis* spores in vitro, the first study in the literature to do so. Further research is required to elucidate the precise mechanisms of action and to evaluate the therapeutic efficacy of these compounds in preclinical and clinical settings. However, the goal of PROP-related studies should be a better understanding of the mechanism of action, including toxicity, and standardisation of their key active components.

RJ, a natural product, has long been used in traditional medicines, health foods and cosmetics in different parts of the world and is recognised as a very valuable product for human health [16, 90, 91]. RJ is produced by the hypopharyngeal and mandibular glands of worker honeybees and plays a critical role in the development of queen bees and larvae [92]. There are many studies showing that royal jelly has many physiological and pharmacological properties, including antioxidant, immunomodulatory, antimicrobial, anticancer, wound healing, anti-aging, antimutagenic, antidiabetic, estrogenic, antiallergic, antiosteoporotic and anti-tumour effects [17, 18, 90].

Studies have shown that the main components of RJ are water (50–60%), proteins (18%), carbohydrates (7–18%) and lipids (3–8%) [90]. A similar chemical content was found in the RJ sample analysed in our study. In the analysis, the compound called 10-HAD, which is one of the most important quality components for RJ, is a substance that shows antibiotic activity against many bacteria and fungi [90]. The amount of 10-HAD, which is one of the most important quality factors for RJ and should be present at a minimum of 1.40% and above by mass [93], was found to be 2.39%.

One study investigated the insecticidal, antimalarial, antileishmanial and cytotoxic effects of RJ and three major fatty acids (trans-10-hydroxy-2-decenoic acid (10-H2DA), 10-hydroxydecanoic acid (10-HDAA), sebacic acid (1,10-decandioic acid)). As a result, RJ, 10-H2DA, 10-HDAA and sebacic acid showed antileishmanial activity. In the study, different concentrations of RJ, 10-H2DA, 10-HDAA and sebacic acid significantly increased nitric oxide production, plasma membrane permeability and caspase-3-like activity levels as a dose-dependent response [94]. In another study, the potential therapeutic effects of combinations containing PROP, RJ, ostrich oil and A. vera were evaluated in BALB/c mice in a model experimentally induced by *L. major*, and the proposed natural combinations were found to be significantly more effective than the standard drug Glucantime^®^ in reducing ulcer size [95]. In our study, we think that 10-HAD may be effective in the strong antimicrosporial effect of RJ. However, further research is required to confirm the effectiveness of this compound and to elucidate its mechanism of action.

## Conclusions

In conclusion, our study, as the first evaluation in the literature, showed potent antimicrosporidial effects of PROP and RJ against *E. intestinalis* in an in vitro model. Due to their potential medicinal and pharmaceutical properties, these natural bee products should be considered as an option in the drug pool for the development of antimicrosporidial drugs. Further research is required to elucidate the precise mechanisms of action and to evaluate the microsporidial activity of bee products in preclinical and clinical settings.

## Data Availability

• The study is the first to evaluate the effect of PROP and RJ on E. intestinalis.• All concentrations evaluated in the study showed a significant inhibitory effect on the growth of E. intestinalis spores compared to the control group.• PROP and RJ are promising products for the development of alternative treatments against microsporidial infections.
